# A Robust Approach to TDMA Synchronization in Aerial Networks

**DOI:** 10.3390/s18124497

**Published:** 2018-12-19

**Authors:** Luis Ramos Pinto, Luis Almeida

**Affiliations:** 1 Instituto de Telecomunicações, R. Dr Roberto Frias, 4200-135 Porto, Portugal; 2 CISTER Research Centre, R. Alfredo Allen 535, 4200-135 Porto, Portugal; 3 Faculdade de Engenharia, Universidade do Porto, R. Dr Roberto Frias, 4200-135 Porto, Portugal

**Keywords:** multi-hop, network, synchronization, TDMA, WiFi

## Abstract

Unmanned Aerial Vehicles (UAVs) can be a powerful tool for live (interactive) remote inspection of large-scale structures or areas of interest. Instead of manual, local, and labor-intensive inspections, we envision human operators working together with networks of semi-autonomous UAVs. The current state-of-the-art for low-delay high-throughput inter-vehicle networking relies on Time-Division Multiple Access (TDMA) techniques that require accurate synchronization among all network nodes. In this paper, we propose a delay-tolerant synchronization approach that converges to the correct order of the TDMA slots implemented over COTS WiFi in a fully-distributed way and without resorting to a global clock. This highly flexible solution allows building an ad-hoc aerial network based on a backbone of relaying UAVs. We show several alternatives to achieve this synchronization in a concrete aerial network and compare them in terms of slots’ overlap, throughput, and packet delivery. The results show that these alternatives lead to trade-offs in the referenced metrics. The results also provide insight into the delays caused by buffering in the protocol stack and especially in the WiFi interface.

## 1. Introduction

Unmanned Aerial Vehicles (UAVs), in particular multirotors, can be used for a myriad of applications such as live (interactive) remote inspection of large-scale structures, e.g., towers, bridges, pipelines, or of specific areas of interest, e.g., for search and rescue [1] or wild-life surveys [2]. In this case, manual, local, and labor-intensive inspections by human operators can be facilitated with the use of semi-autonomous UAVs, which can be done in three stages.

Firstly, an operator at a ground Base Station (BS) defines a remote Area-of-Interest (AoI) and instructs a group of semi-autonomous sensor-capable UAVs to navigate there; secondly, interactive control of the fine position and pose of UAVs is initiated to focus on features of interest; concurrently, a live stream of sensor data from the AoI to the BS is initiated; finally, the necessary communication backbone is established, by means of a complementary autonomous group of relaying UAVs, linking sensor UAVs to the BS.

**Figure 1:**
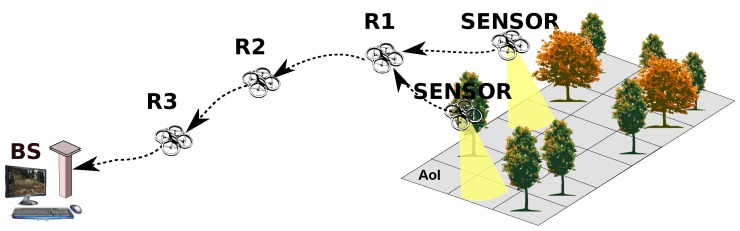
Our vision of an aerial multi-hop sensor network, streaming to a base station through a backbone of relaying UAVs.

This vision, illustrated by Figure 1, is accompanied by the perception that the current state-of-the-art in UAVs still misses efficient solutions for creating and maintaining a high throughput backbone network [3]. While some works do address the problem of maintaining a multi-hop topology, possibly a line, permanently connected by controlling nodes’ movements [4,5], the case of high-throughput streaming adds further concerns on queuing, packet loss and mutual interference. Unfortunately, these aspects are often overlooked in the current UAV literature.

In the related field of networked mobile robots, robots’ motion can be controlled to maintain high-throughput for streaming data to a base station. In [6], the authors proposed concentrating transmissions in areas where/when the channel is good, slowing the robot, and then moving faster in areas with poor channel characteristics. Conversely, the mobility of our nodes is driven by sensing, and thus, we aim at making the best of the current channel, instead. Other works leverage delay-tolerant networking. For example, the authors of [7] proposed switching the robots to a data-mule mode when they move far from the base, giving away the live connection, which is incompatible with our vision.

### 1.1. Contributions

We claim that user-interactive area coverage and inspection applications relying on UAVs, without the support of a pre-installed networking infrastructure, need a communication backbone properly setup to achieve reliability, a long range, high throughput, and low latency. These requirements arise from the use of video monitoring, as presented in our vision, and its use for effective control of sensing drones through video feedback.

The difficulty of setting up such a backbone arises from the instability of the links, their asymmetry, i.e., differences among each other, and the high traffic associated with video forwarding, which, altogether, give rise to backlogs due to traffic accumulation and potential information loss [8]. As such, coordination of transmissions among several nodes is vital to prevent backlogs and their associated delays from growing beyond acceptable.

Time-Division Multiple Access (TDMA) is an old coordination technique [9] that grants all nodes an exclusive periodic transmission window, called a slot, thus preventing phenomena like starvation and mutual interference in channel access.

Therefore, we have proposed recently a novel framework that creates an overlay TDMA structure over CSMA/CA (MAC used by the WiFi standard), joining the benefits of both. In [10], we proposed a model that related reliability with link-length for a slotted multi-hop line network, producing optimal relay positions with symmetrical links. In [11], we showed that keeping slots constant when the links are asymmetrical leads to buffer overflows and high packet losses, and we proposed the distributed variable slot-length protocol that maximizes throughput while minimizing latency. Alternatively, we proposed the dynamic relay placement protocol [8] that adjusts the relay positions to equalize links’ reliability.

However, our previous works left the TDMA synchronization as an open issue. This is the problem we are now addressing, towards setting up a TDMA-based communication coordination on an aerial backbone network, and we put forward three contributions to the-state-of-the-art, namely:

1. A new method to implement TDMA on devices with COTS WiFi interfaces.
2. A novel distributed and adaptive mechanism to synchronize nodes online on a multi-hop aerial network without a global clock.
3. Comparative evaluation on a concrete UAV platform.

The comparison will focus on different alternatives to achieve the desired synchronization, making different uses of the delay information that can be extracted from each packet in each link.

### 1.2. Organization

The rest of the paper is organized as follows. The next section provides the background and state-of-the-art on TDMA solutions for wireless networks. In Section 3, we formulate our problem, and in Section 4, we explain how to implement a TDMA mechanism on (potentially) any wireless device without modifying its core (neither OS nor drivers). Section 5 exposes our novel distributed synchronization methods. To evaluate our novel methods, we perform an experimental evaluation, starting by the methodology in Section 6 and showing the results in Section 7 obtained from a concrete multi-hop network of UAVs, which we discuss thoroughly in Section 8. Lastly, Section 9 provides final remarks.

## 2. Background and Related Work

**Figure 2:**
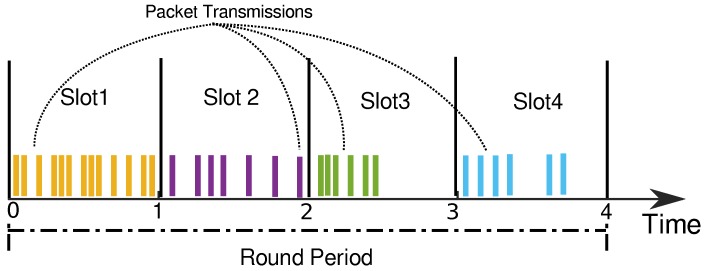
TDMA allows each node to send packets in its own slot. Each of the vertical colored bars represents a packet transmission.

TDMA is a mechanism to multiplex in time the access to a shared resource, assigning to each contender a dedicated slot for its own use. Thus, TDMA is contention-free. In the case of a network relying on a shared medium, such as Radio Frequency (RF), TDMA provides a separate slot to every transmitter in the network, preventing mutual interference. The sequence of slots assigned to all transmitters is typically called a round, and it is endlessly repeated with a given period, i.e., the round period. Without loss of generality, we will consider all slots to be similar, as Figure 2 illustrates.

The round period is typically constant and represents the periodicity at which each node can transmit, i.e., the slot period. For this reason, TDMA imposes delays in the access to the network due to the relatively large periods of inaccessibility, waiting for the right slot. These are more noticeable under light traffic conditions. Conversely, under high traffic loads, the structure of transmissions in exclusive slots has a positive effect of avoiding mutual interference, and the delays grow as a function of the slot width and the local traffic generation, only.

This behavior is essentially opposite that of the widely-disseminated access protocol called Carrier-Sense Multiple Access (CSMA), according to which nodes sense the medium and transmit immediately if free or wait if busy. When access collisions occur, an arbitration mechanism enters in place, typically based on backing off and retrying later. Notably, the WiFi standard uses a variant of CSMA that includes a proactive random waiting mechanism to reduce the chances of access collisions, being thus known as CSMA/CA, where CA stands for Collision Avoidance.

Many discussions and comparisons were made to date between TDMA and CSMA/CA. The work in [12] analyzed the behavior of multi-hop networks under TDMA versus CSMA/CA. This work addresses networks in general, focusing on a small-scale case, and the authors conclude that, depending on payload size and slot length, both medium access control techniques can dominate one another in terms of worst-case network delay.

Attempting at combining the best of both techniques, several works use TDMA implemented over CSMA/CA [13,14]. TDMA reduces mutual interference under high traffic loads, while CSMA allows coping with packets transmitted outside the slots’ structure, either due to poor synchronization, or because they convey urgent events, or because they are external to the team, i.e., alien traffic.

A line of protocols that rely on overlay TDMA is the so-called RA-TDMA for Reconfigurable and Adaptive TDMA. Three of these protocols have been developed over the years, the original RA-TDMA working on infra-structured networks  [15], RA-TDMA+ working on ah-hoc mesh networks [16], and RA-TDMAp specifically tuned to platooning [17]. They are all meant to share a state among a set of autonomous agents, typically achieved with the periodic transmission of a single packet. Moreover, they account for the delay incurred by such a single packet to synchronize and converge to a temporally-consistent notion of round and its slot structure.

In the general case of TDMA implementations, the round consistency among the involved nodes is achieved with clock synchronization [18]. This can be enforced with an external high precision periodic signal that is transmitted out of band and that reaches all nodes at the same time, except for distance-induced differences in propagation delay [19], but this requires a suitable extra hardware that might not be available in COTS interfaces. For example, the Pulse Per Second (PPS) signal that some GPS receivers provide is not always available, as is the case of our platform.

Clock synchronization can also be achieved exchanging messages in the same network, e.g., using Network Time Protocol (NTP) or Precision Time Protocol (PTP) standard protocols [20,21]. However, the mobile and ad-hoc nature of the aerial networks we are considering makes standard protocols perform poorly due to links being asymmetric and volatile and to asymmetric routing paths among the nodes with highly variable forwarding delays, as explained in [18]. This problem could be attenuated using dedicated hardware to timestamp packets with higher precision, as typically used with PTP, a solution that is not available in our platform. It could also be attenuated in some technologies, like IEEE 802.15.4 spanning tree networks, with the Glossy solution [22], which makes use of constructive interference among packets resent by relays, but this is not yet applicable to WiFi and is not efficient in line topologies.

In our work, for the sake of robustness and simplicity of deployment, we opted for a synchronization approach similar to that of the RA-TDMA protocols, i.e., synchronizing accounting for packets’ delays. In this approach, the TDMA layer becomes self-sufficient with respect to synchronization, becoming aware of interference patterns, as well. Thus, it can adapt to escape from pernicious patterns such as alien traffic transmitted with the same period of the TDMA framework. However, RA-TDMA protocols did not consider an on-line stream of sensor data; packets were broadcast and sent scarcely, not generating queuing issues.

Therefore, to the best of our knowledge, how to use the packets’ delays, under high traffic loads, for automatic round synchronization is still an open issue that we tackle in this work. This is a robust approach since it tries to mitigate the impact of physical delays affecting the packets by actuating on the phase of the time window in which a node can transmit.

Finally, our solutions to synchronize nodes in an ad-hoc TDMA structure are orthogonal to, and compatible with, many other RF solutions and protocols that can improve communication on a multi-hop network, such as channel re-utilization, parallel use of orthogonal channels and directional antennas for Signal-to-Noise Ratio (SNR) improvement.

## 3. Problem Statement

In this work, we consider a line network with *n* nodes willing to transmit a significant traffic load. For simplicity, we consider only one sensor node at one tip of the line, which is the streaming source (multiple sensor nodes could be easily accommodated, sharing the connection to the first relay and with an aggregated transmission requirement similar to that of a single sensor node). The stream has to go through *n* hops to reach the base station, each hop adding a non-negligible forwarding delay and, thus, impacting significantly the end-to-end latency. For this reason, we consider *n* to be limited to low values, say up to five. Beyond this limit, we experienced end-to-end latencies in the seconds scale [11], turning the control of the sensor UAV impractical by an operator in the base station using the sensor streaming.

The line topology is set up releasing or removing the relay UAVs as needed to support the backbone network [10]. Relay UAVs are guided autonomously to take adequate positions in the backbone, e.g., using GPS. Note that the physical topology can be a mesh, including more links than the backbone line, and thus, the line topology is enforced logically with routing, reconfigured every time a relay is added or removed.

Each node is assigned a unique slot, characterized by a unique, sequential ID along the line, from 1–*n*, and a time span, also known as slot-length *s*. Node *i* is assigned slot $s_{i}$. A node is allowed to transmit any en-queued packets during this time span, every *T* units of time, i.e., the TDMA round period. The offset with which all packets are transmitted is piggybacked in the packets and transmitted together with their payload.

The main problem implementing such a system arises from the distributed nature of the wireless mobile network [18]. Each node has its own clock, and the reference of time is not absolute. Without proper synchronization, nodes will set overlapping slots and transmissions, creating mutual interference.

Another problem arises from the implementation of an overlay protocol over WiFi. The last control event when transmitting a packet is when it is submitted to the Network Interface Card (NIC). After this moment, there is no information on when exactly a packet is sent to the medium due to the possibly busy medium, access collision, packet loss and retransmission, etc. Consequently, it is always possible that packets arrive later than expected. The situation is worsened when submitting several packets to the NIC in a row, to increase bandwidth efficiency.

Therefore, we define the following two research problems:

- Within its own local time frame, how can a node determine when is the beginning of its slot, using simply its ID, the offsets’ information conveyed in the packets, and their reception instants?
- When should a node, within its slot, stop transmitting to increase the chances that its packets will still be received inside its slot?

## 4. TDMA Overlay Method

As we explained in Section 2, we will set up a TDMA layer on top of COTS WiFi interfaces, using local time, only, while synchronizing slots in a logical manner, using the delays suffered by the packets. Notice that CSMA/CA is still in place, handling any out of time transmissions that may occur. In this section, we will show how each node sets up its own transmission slot.

**Figure 3:**
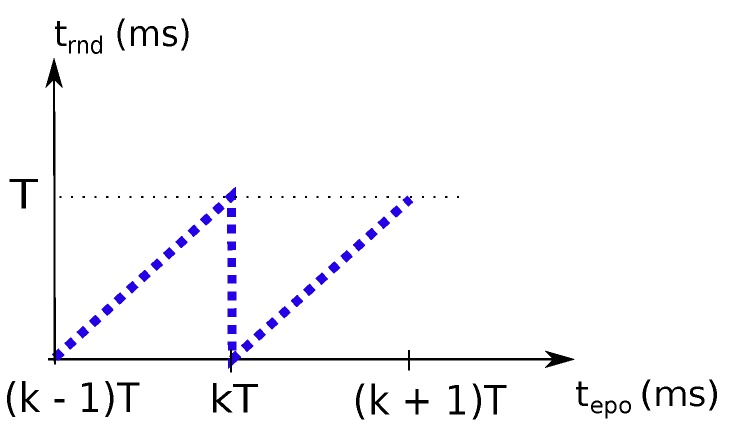
Round-time $t_{rnd}$ is a modulo *T* representation of epoch time $t_{epo}$.

Each node has an internal clock, from which the current epoch time is extracted ( i.e., the number of seconds since 1 January 1970) with nanosecond resolution. Thus, clock time is converted to milliseconds yielding $t_{epo}$, and it is used to compute local current round-time $t_{rnd}$, which is given by the modulo operation with the TDMA round period *T* (in ms). This operation is depicted in Figure 3. Since $t_{epo}$ is a fractional number, we refine the classical integer modulo operation, yielding Equation (1). Notice that, without clock synchronization, each node will have its own $t_{epo}$ and $t_{rnd}$ frames.

(1) trnd=tepomodT+frac(tepo)wherefrac(x)=x−xtrnd∈[0,T[,xT∈N

**Figure 4:**
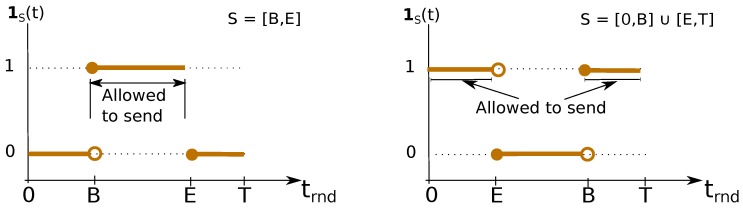
A TDMA transmission slot *S* is defined by its boundaries *B* and *E*. A node is allowed to transmit packets if current round-time $t_{rnd}$ is within *S*, i.e., if $1_{S}\left(t_{rnd}\right)=1$.

We assume that round period *T* is a positive integer, constant, and known in advance. Each node is assigned one transmission slot *S*, which is defined by both its (constant) ID and (shift-able) boundaries. Slot ID is a unique constant identification, a positive integer, pre-assigned to each node ∈[1,n]. Slot *S* boundaries are defined by beginning and ending time marks: *B* and *E* (∈[0,T[), respectively. *B* is sometimes referred to as the phase of the transmission slot, as it is the time-span the node waits for to start transmitting, since the beginning of its local round-time $t_{rnd}=0$. Note that round-time is cyclic with period *T*; hence, *B* might be greater than *E*, exemplified in Figure 4.

Therefore, we define slot *S* boundaries s.t.:

(2) $$S=\left\{\begin{array}{cc} {}[B,E[ & \mathrm{if}B<E\\ {}[0,E[\cup [B,T[ & \mathrm{if}B>E \end{array}\right.$$

**Figure 5:**
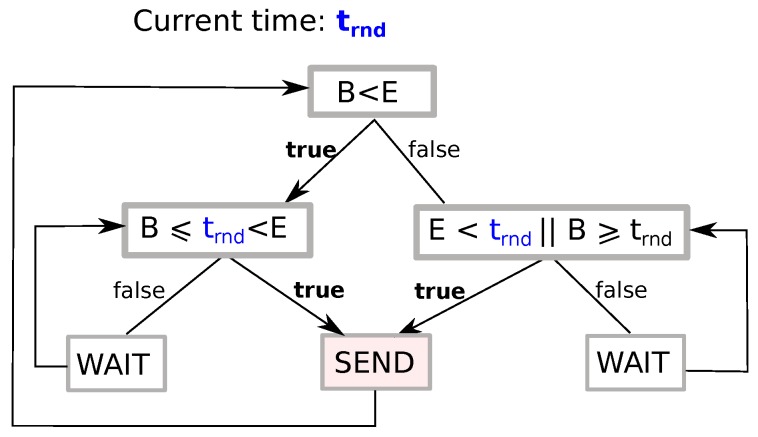
Decision process for queued packets, given *B* and *E*, at time $t_{rnd}$: send or wait? This defines function $1_{S}\left(t_{\mathrm{rnd}}\right)$ for any *S*.

A transmitter node may send queued packets if and only if its local current round-time $t_{rnd}$ is in-between the boundaries of *S*, i.e., if indicator function $1_{S}\left(t_{rnd}\right)$ is equal to one, as shown in Figure 4. These conditions are clarified by means of the fluxogram of Figure 5.

Slot-length *s* is defined has the time-span a node has available to transmit, and it is depicted in Figure 4, as well. It is considered constant and defined by Equation (3) ( for convenience of notation, we define the operator ${\left(x\right)}_{T}\equiv \left\{\left(x+kT\right)\;\mathrm{mod}\;T\right\}$, where $k\in N_{0}:\left(x+kT\right)\geq 0$).

(3) $$\begin{array}{cc} s & ={\left(E-B\right)}_{T} \end{array}$$

## 5. Synchronization Methods

In order to synchronize slots, we have designed a general mechanism where nodes shift their own slot boundaries as needed to avoid overlapping transmissions. We follow an approach similar to that used by the RA-TDMA family of protocols, but now tuned to the case of high traffic loads in aerial backbones, as needed for aerial video sensing.

The synchronization method can briefly be explained as follows. A (receiver) node shifts its own slot boundaries by analyzing the delay of each incoming packet. If, from the receiver’s perspective, packets are arriving later than expected, the receiver shifts its own slot and thus starts transmitting later to avoid overlapping transmissions. On the other hand, if, from the receiver’s perspective, packets are arriving earlier than expected, then the receiver keeps its own slot as is, waiting for the transmitter to advance its slot. Note that the current transmitter will perceive the packets later on sent by this receiver as being late.

Therefore, all nodes will shift their own slots boundaries until no delay is detected, converging to a situation of no or minimal overlapping between slots. Convergence can be proven in the same way as it was for RA-TDMA+ [16].

### 5.1. Algorithm

We will now detail how to synchronize two nodes, namely node *i*, the transmitter, and node *j*, the receiver. The first step is to compute the delay of every received packet *k* ($\delta_{k}$). It is estimated by the difference in the round-time of the receiver between the actual receiving instant (${\mathrm{rx}}_{k}$) and the expected time of arrival of that same packet (${\hat{\mathrm{rx}}}_{k}$), s.t.:

(4) $$\delta_{k}={\left({\mathrm{rx}}_{k}-{\hat{\mathrm{rx}}}_{k}+T/2\right)}_{T}-T/2$$

${\left(x+T/2\right)}_{T}‒T/2$ guarantees that delay *x* either positive or negative is centered around zero. Given the distributed nature of the synchronization, $\hat{\mathrm{rx}}$ computation is not trivial. The receiver node, with slot ID *j* and boundaries $B^{\left(j\right)}$ and $E^{\left(j\right)}$, needs to acquire two metrics within the header of an incoming packet *k*, namely:

1. Transmitter’s slot ID (*i*): we assume this is immutable along the mission and set adequately to minimize end-to-end delay of the sensing stream, i.e., the source will have ID 1 and then sequentially until the last relaying UAV that will have ID *n*.
2. Message position ($p_{k}$); this is the transmission offset, in the transmitter’s round-time frame, relative to the beginning of its slot.

Thus, let us assume all *n* slots have the same length *s* and that the node with slot ID *j* was perfectly synchronized with its neighbor (node *i*). Then, node *j* would expect the neighbor’s beginning boundary ${\hat{B}}^{\left(i\right)}$ to be given by Equation (5).

(5) $$\begin{array}{c} {\hat{B}}^{\left(i\right)}={\left(B^{\left(j\right)}-\left(j-i\right)s+T\right)}_{T}\\ \mathrm{where}\;j,i\in [1,n]\;\mathrm{and}\;s=T/n \end{array}$$

**Figure 6:**
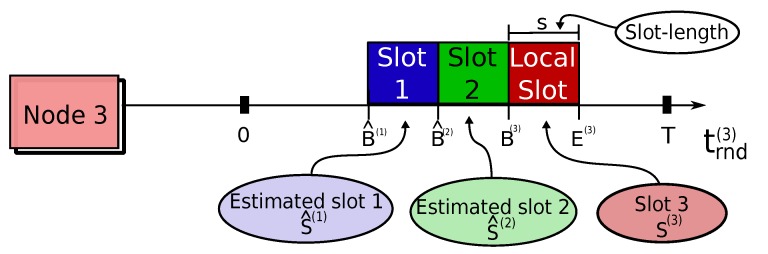
The node with slot ID 3 (red) estimates boundaries of slots ID 1 (blue) and ID 2 (green), by using Equation (5).

This process is exemplified in Figure 6; the receiver node, with slot ID 3 and slot boundaries $S^{\left(3\right)}=\left[B^{\left(3\right)},E^{\left(3\right)}\right]$ (red), estimates the initial boundaries of slots ID 1 (blue) and ID 2 (green) to be ${\hat{B}}^{\left(1\right)}$ and ${\hat{B}}^{\left(2\right)}$, respectively.

If nodes were perfectly synchronized and propagation time negligible, a receiver *j* would expect the time of arrival of a given packet-*k* from slot *i* to be $r{\hat{x}}_{k}$ (Equation (6)).

(6) $${\hat{rx}}_{k}={\left({\hat{B}}^{\left(i\right)}+p_{k}\right)}_{T}$$

Then, combining Equations (4) and (6) allows computing $r{\hat{x}}_{k}$, yielding Equation (7).

(7) $$\delta_{k}={\left({\mathrm{rx}}_{k}-{\hat{\mathrm{rx}}}_{k}+T/2\right)}_{T}-T/2={\left({\mathrm{rx}}_{k}-\left({\hat{B}}^{\left(i\right)}+p_{k}\right)+T/2\right)}_{T}-T/2$$

**Figure 7:**
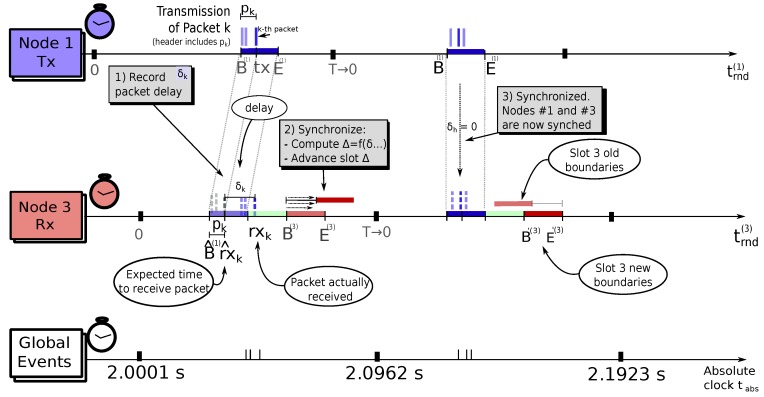
Shifting slots in the TDMA round to enforce synchronization. The upper timeline shows the local time frame of Node 1 as the transmitter, which was slightly advanced (blue slot). The middle timeline shows the local time frame of Node 3 as receiver, which senses Node 1 slot advancement and shifts its own slot (red slot), accordingly, maintaining synchronization. The lower timeline shows the global synchronization events, i.e., packets’ transmissions, on a virtual absolute time frame.

Figure 7 illustrates this synchronization process using the individual timelines of the transmitter and receiver, as well as a global absolute timeline that shows the global events used for the synchronization, i.e., the actual packet transmissions.

Right at the beginning of each transmission slot, the respective owner node analyzes all the packet delay estimates saved during the previous round ($\delta_{1},\delta_{2},\ldots$) to compute an aggregated delay function $f(\delta_{1},\delta_{2},\ldots )$ and resets the packet delay estimates for the next round. Node *j*, then, shifts (advances) the phase of its own slot according to Δ, as shown in Equation (8) and Equation (9). This process repeats every round *r*. For the convenience of representation, we define bound(x,A,B)≡min(max(x,A),B).

(8) $$\Delta (r)=\mathrm{bound}(f(\delta_{1},\delta_{2},\ldots ),\;0,\;\Delta_{\max})$$

(9) $$\begin{array}{cc} B^{\left(j\right)}\left(r\right) & =B^{\left(j\right)}\left(r-1\right)+\Delta \left(r\right) \end{array}$$

The use of the bound function keeps the phase adjustments between zero and $\Delta_{max}$, thus being always positive. This means that slots can only be delayed. Otherwise, we could have strong slots overlapping with potential network transient overloads.

In practice, the delays affecting the packets received in each round are rather variable due to variations of the time spent in outbound and ingress buffers, in the CSMA access and backoff mechanism, and by the interference of operating system mechanisms. Therefore, there are multiple options for the correction function (*f*) to combine such variability and compute the synchronization error. In this work, we will study three different correction methods, each with its own properties.

These methods are different from that used in the RA-TDMA protocols, in which every node sends a broadcast packet at the beginning of its own slot. There, a receiver node computes the maximum delay of all received packets and uses that for the phase compensation.

Our methods rely on information piggy-backed at every packet a node receives, from any neighbor, but packets are all unicast. The methods we considered are the following:

- **[MIN]** Minimum delay: f(·)≡min(·) uses the packet with the lowest estimated delay in the set. This means our local slot will advance the least.
- **[MAX]** Maximum delay: f(·)≡max(·) takes the opposite approach and advances the local slot the most, trying to compensate for the longest observed delay.
- **[MED]** Delay’s Median: f(·)≡median(·) considers all observed packet delays and mitigates the impact of outliers using the median of the sample.

### 5.2. Remarks

In the event that no packet is received during one round, the slot owner holds its slots still and no adjustment is done. In cases of high traffic load, as our target use case, this should be a rare case.

On the other hand, all methods consider the parameter $\Delta_{\max}$, set at configuration time, to bound the maximum phase adjustment that can be applied to a slot in any round and thus avoid unbounded jumps in slot position. This effectively controls the slot adjustment process, accelerating, if larger, or slowing, if smaller, the convergence to a global virtual TDMA round. The impact of $\Delta_{\max}$ is similar to the corresponding parameter in RA-TDMA+, studied in [16]. The effective period of this virtual round is perceived independently by each node ($T^{\left(j\right)}$) as the time lapse between the beginning of its own slot in the current (*r*) and previous rounds (Equation (10)).

(10) $$\begin{array}{cc} T^{\left(j\right)}\left(r\right) & =B^{\left(j\right)}\left(r\right)-B^{\left(j\right)}\left(r-1\right)+T=\Delta \left(r\right)+T\\ T^{\left(j\right)}\left(r\right) & \leq \Delta_{\max}+T \end{array}$$

Moreover, note that packets can occasionally suffer delays that are larger than $\Delta_{max}$, potentially falling into the following slots, thus overlapping with the transmissions of other nodes. This overlapping is sorted out with the native WiFi CSMA/CA arbitration that attenuates the potential increase in packet  losses.

This feature allows our protocol to operate in open environments and with some degree of synchronization error. Interference caused by other traffic, potentially alien, and by hidden nodes will appear as delays affecting packets, triggering the slots’ adaptation process. As a result, our protocol will move the phase of the global virtual TDMA round to an offset that nullifies interference, if that is possible, or else will continue shifting the phase with an effective period larger than *T* until the interference is over. In such situations, given that the slot length *s* is kept constant, the effective channel available bandwidth is reduced to between s/T and $s/(T+\Delta_{max})$. Our protocol adapts seamlessly to all situations that can occur in the channel that lead to delays in the data packets.

Finally, we remark that our protocol also bears similarity with Lamport clocks and the Happened-Before (HB) relationships [23]. This is natural since we are aiming at enforcing the correct slot order. However, the limitation on the slot-shifting correction that is applied each round ($\Delta_{max}$) also limits the drift rate between our virtual time and physical time, which goes beyond typical logical clocks.

## 6. Evaluation Methodology

To evaluate the relative merits of the proposed synchronization methods, we defined a set of five metrics that will be applied to an experimental campaign with an actual UAV backbone.

The metrics are computed on a round-period basis and will be shown in the Results Section. The first two are synchronization error and overlap ratio and regard one link in particular, i.e., two connected nodes. The next one is the effective period of the global virtual TDMA frame, and it can be observed by any node. The last two regard the full network end-to-end and are the throughput and the packet delivery ratio (PDR). These can also be computed at the link level.

### 6.1. Synchronization Error $\epsilon_{S}$

This metric evaluates how tight the synchronization on a given link is. At the beginning of each slot of a given node (local slot), we measure the time-span between the (estimated) end of the (preceding) neighbor’s slot and the beginning of the local slot. Ideally, this is zero. A negative value means the previous slot is advanced and finished earlier than expected, creating a gap. Conversely, a positive value means the previous slot is late, creating an overlap. This metric implies an estimation of the current neighbor slot, thus it is an indirect metric.

The synchronization error $\epsilon_{S}$, for node *j* in round *r*, is computed by the difference between the estimated end of the neighbor slot j‒1 and the beginning of local slot *j* (Equation (11)).

(11) $$\epsilon_{S}^{\left(j\right)}\left(r\right)={\hat{E}}^{\left(j‒1\right)}\left(r\right)‒B^{\left(j\right)}\left(r\right)=\left({\hat{B}}^{\left(j‒1\right)}\left(r\right)+s\right)‒B^{\left(j\right)}\left(r\right)$$

### 6.2. Overlap Ratio $\epsilon_{O}$

This metric evaluates overlapping packets, i.e., it accounts for the percentage of packets received at a node during its local transmission slot with respect to the total number *m* of packets received during the preceding round *r* (Equation (12)).

(12) $$\epsilon_{O}^{\left(j\right)}\left(r\right)=\frac{1}{m}\sum_{k=1}^{m}1_{S(r)}^{\left(j\right)}\left({\mathrm{rx}}_{k}\left(r\right)\right)$$

This is a direct metric since it does not depend on estimations or assumptions of neighboring nodes, but solely on measurements done directly by the node.

### 6.3. Effective Period $\epsilon_{T}$

This metric coincides with $T^{\left(j\right)}\left(r\right)$ measuring the effective period of the global virtual TDMA frame as seen by node *j* considering its local slot (Equation (13)).

(13) $$\epsilon_{T}^{\left(j\right)}\left(r\right)=T^{\left(j\right)}\left(r\right)$$

The effective period also says how much network bandwidth is available in the global virtual TDMA frame and how precise this frame is. The closer it is to the round period *T*, the higher the bandwidth available and the more precise the global time frame is. Larger delays imply larger adaptations that will increase the effective period, degrading both bandwidth and precision.

### 6.4. End-to-End Throughput $\epsilon_{Th}$

This metric measures the actual throughput that the network is providing to the sensing application as seen by the BS. It is measured by the amount of data in bytes *D* received at the BS during round *r*, divided by the round length *T* (Equation (14)).

(14) $$\epsilon_{Th}\left(r\right)=\frac{D(r)}{T}$$

### 6.5. End-to-End Packet Delivery Ratio $\epsilon_{P}$

This metric measures the ratio of packets effectively transferred end-to-end, i.e., the ratio between the packets D(r) received by the BS over the total number of packets M(r) sent by the source node in round *r* (Equation (15)).

(15) $$\epsilon_{P}\left(r\right)=\frac{D(r)}{M(r)}\in \left[0,1\right]$$

### 6.6. Setup

All synchronization methods were implemented in a set of AR Drone 2.0 UAV platforms, each encompassing an ARM 700-MHz CPU with an 802.11 g embedded-card operating at 24 Mb/s, and MAC retries set at two. This retry value was chosen to contain the variation of the duration of a single message transaction. It was, in fact, the smallest value our platform allowed. Larger values may create wide variations in message transmission times that can pervert any timed transmission control, especially when the channel degrades. This is a good motivation to use all packet delay information for our synchronization, to mitigate possible outliers. Concerning the fixed transmission rate of 24  Mb/s, the reason is similar. An adaptive transmission rate improves reliability, but degrades timeliness. The chosen value is known to be a good compromise [24].

The topology of the network has three drones, each with one assigned slot, plus the Base Station (BS), creating a line network of three hops. Drone 1 is the source of data (video stream), while the BS is the respective receiver. Intermediate nodes are in charge of relaying data, forming an aerial backbone that extends the communication range of the source node.

The size of three nodes, with a backbone of two relay drones, is realistic, given the limitations on acceptable end-to-end latency for effective visual feedback to control the position of the sensor drone using its own video stream at the BS.

The position of the drones was fixed in 3D, with 3 m of separation, inside a laboratory, to improve the repeatably of the experiments, controlling most of the external variables. This allows focusing on testing and evaluating the synchronization methods without the interference of motion-related issues, e.g., variable GPS precision and autonomous navigation.

The physical topology was a mesh with all nodes within the range of each other. This fully connected topology recreates a worst-case scenario concerning mutual interference when comparing to a typical operational scenario with nodes far apart, since all nodes hear each other, increasing channel utilization and the potential for triggering the CSMA back-off mechanism.

The round period is T=96 ms; slots are s=32 ms long; and slot IDs are in ascending order from the source toward the BS to minimize latency. The source sends one frame roughly every 130 ms (7.5 FPS), each frame with 11 KB divided into 73 UDP packets with a 154-B payload. We use small packets to increase their reliability and to stress the packet management of our protocol.

In the opposite direction, there is a default beacon packet with commands from the BS to the source node sent by the BS every 48 ms without a slot. When forwarding these packets, all relaying nodes communicate bidirectionally, which allows closing the round having the last slot influencing the position of the slot of the source node. The slot phase adjustment is limited in each round to $\Delta_{max}=8$ ms (i.e., 25% of the slot-length).

Finally, we piggyback a TDMA header with nine bytes in each packet to allow packet delay estimation. This header has the following structure:
uint8_tslot_id; // 0–254 (one slot per host)slot_limits_tslot; // [init end] ms - 16bitsuint8_ttimestamp_ ms; // when we call sendto()uint8_ttimestamp_part ms; // # of parts in 256 - fraction of 1 msuint32_tseq_num; // unique packet id

This is the only communication overhead, which is negligible. In terms of computations, packet delay handling implies simple arithmetic, only, and the aggregate delay function (**MIN**, **MAX**, and **MED**) is computed on typically less than 60 packets per round. These operations also incur a negligible overhead. As we will see in Section 8.3, the overhead of our transmission control approach arises from the way data are passed to the NIC, i.e., one packet at a time. Despite potentially reducing maximum throughput, this is the price to pay for improved timeliness.

## 7. Results

This section shows the results obtained after running the setup presented in the previous section for approximately 5 min (over 3000 rounds) with each synchronization method. The results are histograms that show the distribution of each metric along the 3000 samples, using a different color for each one of the synchronization methods. The histograms were generated with the hist(Samples,x) MATLAB function. The values inside the hovering boxes show the average of those 3000 samples for each method.

### 7.1. Synchronization Error

**Figure 8:**
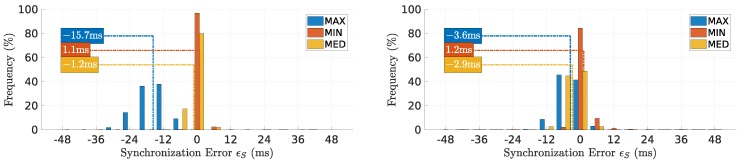
Distribution of synchronization error $\epsilon_{S}$ on the first and second relays, left and right respectively. **MIN** shows the smallest synchronization error with a light overlap. **MED**, delay’s Median.

Figure 8 shows the distribution of synchronization error $\epsilon_{S}$ on the first and second relays for all rounds. **MIN** succeeded in keeping the synchronization error close to zero despite a tendency to create a small overlap of near 1 ms. This is expected, given the use of the smallest delay to compensate the slot position, thus not escaping completely from delays affecting the previous slot.

Conversely, the **MAX** method showed a larger variability of synchronization error values, which was inherited directly from the variability of the maximum packets delay that can reach significant great values. This led to frequent large compensations that moved a slot away from the previous, leading to frequent gaps that were particularly large in the first link, reaching an average of 15.7 ms. On the other hand, the **MED** method ignored both early and delayed packets, achieving a synchronization with a few cases of overlap, with the average showing a small gap of 1.2 ms and 2.9 ms in the first and second links, respectively.

### 7.2. Overlap Ratio

**Figure 9:**
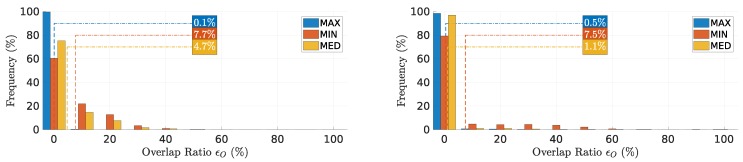
Overlap ratio $\epsilon_{O}$ on the first and second relays, left and right, respectively. **MAX** exhibits almost no overlapping packets.

Figure 9 shows the distribution of overlap ratio $\epsilon_{O}$ for the first two links as in the previous case, for all rounds.

The **MAX** method showed most of the rounds without any overlapping packets (near 0%). **MIN** showed higher overlapping packets (average of 7.6%), despite a different distribution in both links. **MED** showed an asymmetric behavior in the two links, with more overlapping packets in the first than the second link. All these results are consistent with those of the previous metric, despite the different measurement approaches (indirect and direct, respectively).

### 7.3. Effective Period

**Figure 10:**
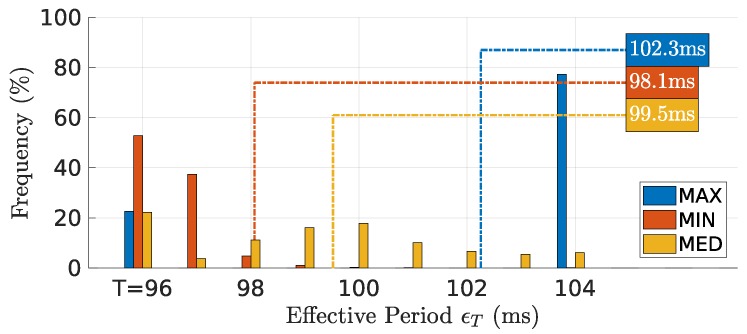
Distribution of the effective period observed by all the nodes. **MIN** results in the least variations of the effective period that remains always close to or on the round period (T=96 ms).

Figure 10 shows the distribution of effective period $\epsilon_{T}$ of the global virtual TDMA frame observed by the source node, for all rounds. The **MIN** metric, given the small phase adjustments, exhibited higher stability of the global virtual TDMA frame, leading to an effective period close to the minimum, from 96 ms (*T*) to 98 ms. **MED** naturally showed a larger spread of adjustments with periods between the minimum of 96 ms and the maximum allowed of 104 ms ($T+\Delta_{max}$). **MAX** showed, most of the time, the effective period on its maximum value of 104 ms, and some times on the minimum value of 96 ms, confirming the larger values that maximum delays can exhibit.

### 7.4. End-to-End Throughput

**Figure 11:**
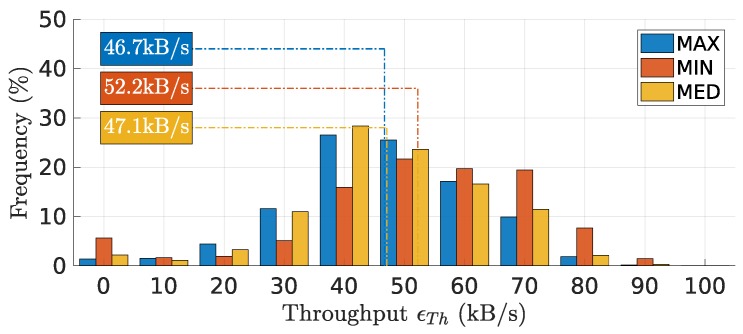
Distribution of the end-to-end throughput $\epsilon_{Th}$. **MIN** has the highest throughput (at 52.2 kB/s) due to the smallest effective period.

Figure 11 shows the distribution of end-to-end throughput $\epsilon_{Th}$ observed during the experiments. All methods exhibited roughly a bell shape, with a peak close to the mean. The fact that **MIN** brought slots closer together, also visible in the smallest effective period, and that the slot length was constant granted it the highest throughput of 52.2 kB/s. For the opposite reason, **MAX** exhibited the lowest throughput with 46.7 kB/s. **MED** showed a value in between, but close to **MAX**, of 47.1 kB/s.

These throughput values are consistent with the peak data rate generated by the source, i.e., 82.5 kB/s = 11 kB × 7.5 FPS. However, this value was reduced differently in the three methods by the transmission control overhead (one packet transmission at a time), exacerbated by the use of rather short packets, and the different effective bandwidth and reliability that each method offers. The total raw capacity of the slotted channel is given by $\frac{s}{T+\Delta_{\max}}\left(\mathrm{PHYRATE}/8\right)$, which with our setup resulted in 920 kB/s.

### 7.5. Packet Delivery Ratio

**Figure 12:**
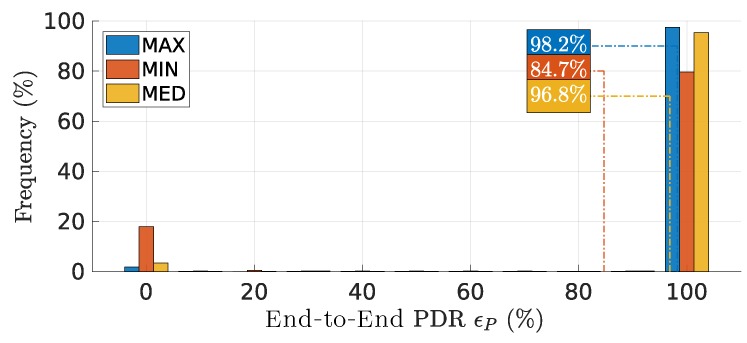
Distribution of end-to-end packet delivery ratio $\epsilon_{P}$ over the mission time.

Figure 12 shows the distribution of end-to-end packet delivery ratio $\epsilon_{P}$ over the mission time. To avoid undefined results, particularly division by zero errors, we ignore all rounds with zero packets sent, thus zero received, as well.

This metric shows clearly the impact of transmissions overlapping in the reliability of the communications. **MAX** had the best PDR due to the lowest packet overlap ratio; **MIN** ’s performance was the worst, with 20% of the rounds delivering no packets, bringing the PDR average down. The PDR at the link level, not shown, also reveals the same behavior

## 8. Discussion

The results shown in the previous section validate the effectiveness of our self-synchronization approach to set up and maintain a global virtual TDMA frame for relaying intense traffic from remote sensing over an aerial backbone network. In this section, we discuss the design guidelines we can infer from the results, and we make other observations related to the timing aspects that we found relevant.

### 8.1. Design Guidelines

In this section, we discuss the results from the point of view of supporting design decisions when setting up an aerial backbone network for remote sensing streaming. The most important result is the capacity of our approach to keep the nodes’ slots separated while accommodating asynchronous traffic, either team traffic that suffered strong delays or alien traffic. Of no less importance, though, is the trade-off between end-to-end PDR, end-to-end throughput, and the stability of the round period.

The three proposed synchronization methods offered complementary behaviors in these aspects. While **MAX** was clearly the option when PDR was the most important aspect, **MIN** would be the best fit if throughput or stability of the round period was the goal. However, the packet overlapping generated by **MIN** caused a degradation in the PDR. This degradation can be even worse than we observed when in the presence of hidden nodes, which are likely to occur when a relay is receiving packets from two neighbors at opposite sides that do not hear each other. Finally, **MED** was shown to be a reasonable compromise, but frequently behaving closer to the worst of **MAX** or **MIN**, thus not being obvious that it is a good choice.

### 8.2. Clock-Drift

Despite the effectiveness of our adaptive synchronization methods, which accommodated large delays, we carried out experiments to assess the behavior of the nodes’ physical clocks. Given that some missions are short, say 5–15 min, we hypothesized that, if the slots are synchronized to a common clock in the beginning, e.g., as provided by an NTP server installed in the BS, maybe the drifts are small enough to allow operation without re-synchronization during the mission time.

**Figure 13:**
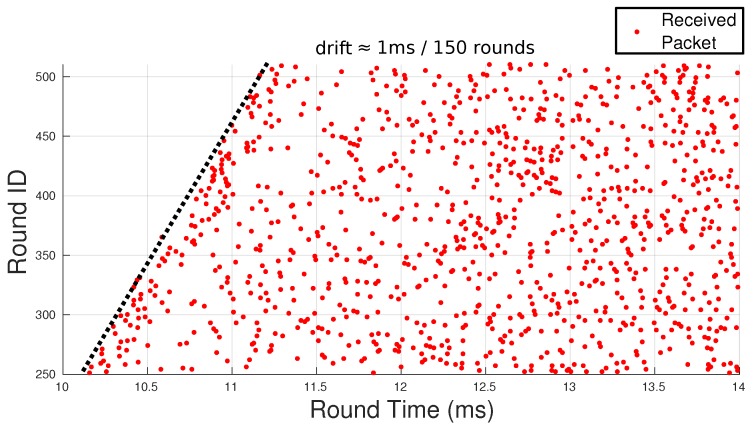
Nodes synchronized using NTP at the beginning of the mission, only. Per line, we can see the reception instants of packets sent by the source in the corresponding receiver slot at the first relay. The transmitter and receiver slots show a clear drift of, approximately, 1 ms every 150 rounds (drift 1:14,400).

Therefore, we ran an experiment without synchronization except at start-up. Figure 13 shows the view of the first relay, representing, per line, the offset of the reception instants of packets sent by the source node, with respect to the start of the receiver’s slot. The drift is marked with the black dotted line on the left, showing the corresponding growing offset with time, with a rate of, approximately, 1 ms every 150 rounds. This is a precision of around $10^{‒5}$ typical of ordinary computer clocks. It also means that, after 5 min (approximately 3000 rounds), the drift would be close to 20 ms, i.e., creating an overlap of slots close to 66%, with a potentially significant degradation in the quality of the communications, e.g., a decrease in PDR, which shows this is not a viable option.

### 8.3. Packet Spreading

One issue that raised our attention due to a negative impact on early experiments was the actual total end-to-end delay at the application level. This is fundamental to control the actual packet reception instants and, consequently, packet overlapping. We realized that packets sent inside the slot-time of the transmitter application layer were received with a rather large delay, thus strong overlapping with the slot of the following transmitter.

**Figure 14:**
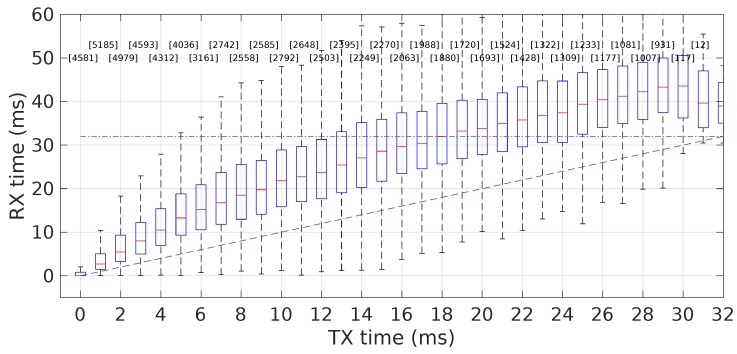
Packet spreading. Packets reception times (*y*-axis) as a function of the respective transmission times (*x*-axis) in the transmitter slot, at the application layer. Reception times grow visibly along the slot, due to packet accumulation in queues.

Figure 14 shows the distributions of packet reception instants as a function of the respective transmission instants at the application layer, both referred to the transmission slot. It is clear that a large component of the end-to-end delay built up in the first 6–8 ms, and then, it still kept growing, but slowly, until the end of the slot. By then, the accumulated delay was already over 12 ms on average, frequently extending to 20 ms or even more. We called this delay accumulation packet spreading.

This effect was caused by insufficient buffer control in the transmitter side that was accepting too much traffic when compared to how much it could send. Consequently, we put in place a tighter buffer usage control at the transmitter, reducing the maximum amount of unsent data allowed in the socket send queue (cf. Linux and SIOCIOUTQ). In particular, we set this limit to 100 bytes, which in fact limits the socket transmit buffer capacity to one video-stream packet at a time.

**Figure 15:**
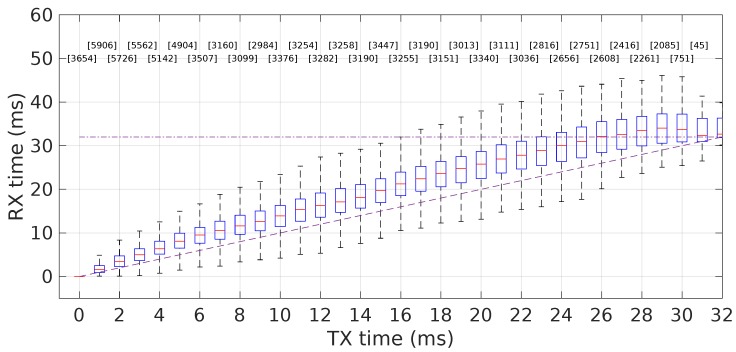
Packet spreading is strongly reduced upon deployment of a transmission control policy at the socket send queue level (maximum amount of unsent data allowed set to 100 B). This policy was used in the experiments reported in the Results Section.

Figure 15 shows the reception times achieved after deploying this transmission control policy. We can see that the end-to-end delays grew significantly less, but mainly, the packets were now essentially delivered within the slot. This mechanism was therefore used in all our experiments shown in the Results Section.

An alternative transmission buffer control mechanism would be to limit the tx-queue length, in the number of packets, at the OS level, setting it to the expected number of packets a node can send during its slot-time. However, in Linux, packets are typically discarded silently when the queue is full, which is an undesirable behavior, and thus, we believe this would be a worse solution.

## 9. Conclusions

Networks of UAVs, particularly quadrotors, are becoming an appealing solution to a myriad of applications, such as remote live monitoring of large structures or areas of interest. In this paper, we proposed a new TDMA protocol particularly suited to support an aerial backbone to connect a remote sensing UAV to a base station. As in any TDMA approach, all nodes are provided a periodic time slot to transmit their data, free of mutual interference. This requires a form of synchronization so that all slots follow one another in a predefined order and desirably without overlap.

In a multi-hop wireless network, especially with a line topology in potentially aggressive environments, clock synchronization performs poorly. Thus, we designed and deployed an overlay TDMA over ad-hoc WiFi that achieves synchronization by compensating the delays actually suffered by the packets during transmission. This is a robust approach since the mechanism copes with all phenomena causing delays in the wireless transmissions. Moreover, the WiFi CSMA/CA access arbitration is still in place, handling late packets and even alien traffic.

In this paper, we presented our synchronization framework focusing on three specific methods to compensate the packets delays, namely based on the minimum, the maximum, and the median of the delays. Then, we carried out an extensive experimental validation that showed that using the minimum grants higher throughput and better TDMA frame stability, while the maximum leads to higher reliability (PDR). The median leads to intermediate performance, but often close to the worst between the other two methods, thus not being very appealing.

Finally, we showed that blind operation based on synchronizing physical clocks in the beginning of the mission is not viable. Moreover, we realized that transmission buffer control was essential to keep end-to-end delays bounded to the transmitters’ time slots, bounding the so-called packet-spreading phenomenon. In the future, we will address channel reuse within this framework to improve overall bandwidth efficiency.
